# Autologous Bioactive Compound Concentrated Growth Factor Ameliorates Fistula Healing of Anal Fistula in a Pig Model and Promotes Proliferation and Migration of Human Skin Fibroblasts via Regulating the MEK/ERK Pathway

**DOI:** 10.1155/2022/7660118

**Published:** 2022-10-14

**Authors:** Xiufeng Zhang, Jianming Qiu, Houdong Wang, Zhenfeng Lu, Shuxian Shao, Jun He, Zhong Shen

**Affiliations:** Department of Coloproctology, Hangzhou Third People's Hospital, Zhejiang, China

## Abstract

Recent evidence suggested that autologous concentrated growth factor (CGF), a new bioactive compound from autologous blood is used widely as an ingenious biomaterial in tissue regeneration with anti-inflammatory properties. This study investigated whether CGF could be involved in the treatment of fistula healing in the anal fistula. For this purpose, the porcine anal fistula model was conducted using the rubber band ligation method and collected pig autogenic CGF to treat the fistulas. CGF treatment promoted fistula healing, which was reflected in the downregulation of inflammatory factors, upregulation of growth factors, and promoted epithelial-mesenchymal transition with increased collagen synthesis. Besides, 16S rRNA gene sequencing analysis of fistula tissues between the control and CGF groups showed that the microbial populations exhibiting significant differences were *VadinCA02*, *Blastomonas*, *Deinococcus*, *Devosia*, *Sphingomonas*, *Rubrobacteria*, and *GW_34*. CGF of volunteers were collected to process small interfering RNA- (siRNA-) ERK or siRNA-negative control transfected human skin fibroblasts (HSF). The results showed that CGF also promoted the proliferation and extracellular matrix-related functions in HSF, as well as activated the MEK/ERK pathway *in vitro* and *in vivo*. Finally, knockdown ERK reversed the effects of CGF in promoting wound healing in HSF. Collectively, our results suggest that the CGF as the bioactive compound from autologous blood exhibited great potential for repairing fistulas as well as promoting the proliferation and migration of human skin fibroblasts by triggering MEK/ERK signaling. These findings provided a fresh perspective for understanding the role of CGF in the management of fistulas.

## 1. Introduction

Anal fistula is a self-growing chronic inflammatory sinus around the rectum and anus [1]. Perianal pain is often accompanied by fever and listlessness, which seriously affects the quality of life of patients with anal fistula [1]. At present, the treatment of anal fistula is mainly surgery, but surgery inevitably causes anal sphincter injury to a certain extent [2]. In recent years, some biomaterial-based minimally invasive treatment methods for anal fistula, which promote the healing of the fistula by injecting biomaterials (bio-glue, anal fistula plug, acellular matrix, autologous or allogeneic mesenchymal stem cells, etc.) into the fistula, hardly cause any damage to the anal sphincter, but these biomaterials still have the disadvantage of the potential risk of allergic reaction with a high cost and have failed in a phase III pivotal trial [3, 4]. Therefore, finding new biological materials with high-cost performance, suitable for promotion and therapeutic effect are warranted.

Autologous platelet-rich plasma (PRP), a platelet concentrate extracted from autologous blood, contains a high concentration of growth factors, which can significantly accelerate wound healing [5]. In addition, PRP has been widely used in clinical practice in recent years due to its simple preparation, high safety, and autologous origin, thus eliminating the rejection between tissues [6, 7]. Moreover, clinical trials have reported that PRP-filled fistula treatment is safe and has a certain cure rate [8, 9]. However, PRP is close to fluid in shape and is difficult to retain in the fistula for sufficient time to affect its role as a biological skeleton. Autologous concentrated growth factor (CGF) is the latest generation of platelet-rich product, which contains high concentrations of growth factors like PRP, but has a more solid structure than PRP [10]. These characteristics make CGF more suitable for the minimally invasive treatment of anal fistula in theory.

Fistula healing consists of three stages: an inflammatory response phase, a granulation phase, and a tissue remodeling phase [11]. In the first stage, white blood cells are recruited to produce a variety of inflammatory factors; in the second stage, recruited fibroblasts, vascular endothelial cells, and epithelial cells participate in angiogenesis and fiber proliferation to induce granulation tissue from the wound [12]; in the third stage, the ECM undergoes recombination, degradation, and resynthesis, with reduced water and blood vessels in the granulation tissue and the formation of scar tissue [13]. In these processes, various cytokines play an important role, including growth factors, inflammatory factors, and matrix metalloproteinases (MMPs) [14]. Among them, epidermal growth factor (EGF) is currently recognized as one of the most closely related cytokines to wound healing [15]. Also, it has been proven that EGF possessed a strong prodivisive activity on a variety of tissue-derived epithelial cells and promoted the migration of epithelial cells, endothelial cells, and fibroblasts [16]. MEK/ERK signaling pathway has been widely validated to be associated with the wound healing process [17, 18]. Extracellular signal-regulated kinases (ERK1/2), the downstream of MEK signaling pathway, are one of the components of the MAPK family [18]. Furthermore, it was found that ERK signaling pathway can promote the growth of granulation tissue in the wound by mediating the proliferation and migration of fibroblasts [19]. Of note, CGF contains a high concentration of EGF which has the ability to activate the ERK signaling pathway [20]. Therefore, we speculated that the injection of CGF into the fistula could activate the ERK signaling pathway. In this study, we sought to determine whether the MEK/ERK pathway was involved in the effect and molecular mechanism of CGF on human skin fibroblast proliferation and migration, as well as fistula healing in a pig model.

## 2. Materials and Methods

### 2.1. Animals and Ethics Statement

A total of 6 male landrace pigs (70-120 days old, 30 kg) were supplied by Wujiang Tianyu Biological Technology Co., Ltd. (Certificate no. SCXK (Su) 2021-0007) and housed in an environmentally controlled room (24°C, 12 h/12 h light/dark, and 50% humidity). Our research was approved by the Animal Ethical and Welfare Committee of Zhejiang Chinese Medical University (Approval number: IACUC-20211108-06, Hangzhou, China).

### 2.2. Construction of the Anal Fistula Animal Model

The pigs underwent anal fistula operation after adaptive feeding for one week. The anal fistula animal model was established as previously described with minor modification [21]. Before surgery, pigs were anesthetized by intramuscular injection of propofol (2 mg/kg). After tracheal intubation, 3% isoflurane (3 L/min) was administered to maintain anesthesia in the pigs. After the anesthesia was effective, routine disinfection was performed, and all the pigs were subjected to anoscopy, digital diagnosis, and anoscopy in the lateral decubitus position. A 0.5 cm incision was made at the lateral margin of the anus about 2 cm from the anus at 3, 9, and 12 o'clock of the lithotomy position (three fistulas were used as control, CGF, and model groups, respectively). A vascular clamp was used to penetrate the anal sphincter structure above the dentate line directly from the small incision. A vascular forceps were used to puncture the anorectal mucosa, and a rubber band was clamped from the anus to penetrate through the whole fistula tract and then exit the perianal incision. The rubber bands were fixed with thin wires to avoid falling off and blocking the anus, and the incision was fixed with a gauze bandage. The pigs were returned to the postoperative care unit until they awoke. After the operation, the vital signs of the animals were detected. The wounds of animals were observed daily, and their dressing changes were made.

### 2.3. Autologous CGF Preparation

10 mL peripheral blood of landrace pig was drawn into a sterile anticoagulation tube and immediately centrifuged in a special machine (Medifuge CGF MF 200100 Silfradent SRL, Sofia, FC, Italy). The centrifugation procedures were as follows: 30s acceleration, 2700 rpm for 2 min, 2400 rpm for 4 min, 2700 rpm for 4 min, 3000 rpm for 3 min, and 36 s deceleration to stop. The middle layer of the centrifuged liquid was used for subsequent experiments.

### 2.4. Groups and Drug Treatment

Four weeks after the operation, the fistulas in the model group were collected for model verification, the fistulas in the control group were subjected to curettage and X-ray examination (X-ray equipment, Philips, Germany), while those in the CGF group were subjected to X-ray examination, curettage, and CGF injection (the fistula was filled, and the suture was performed at the external orifice). Six weeks after surgery, all the animals were further evaluated for fistula healing by B-mode ultrasonography, and the skin on the surface of the fistula was collected for histological staining and immunohistochemistry assays. Eight weeks after surgery, all animals were examined by B-mode ultrasound, and the fistulae were collected for histological staining, immunohistochemistry, enzyme-linked immunosorbent assay (ELISA), quantitative reverse transcription-polymerase chain reaction (qRT-PCR), and Western blot analyses, and the distribution of fistula flora was also identified by 16S DNA sequencing.

### 2.5. B-Mode Ultrasonography Analysis

A proper amount of ultrasonic coupling agent was applied to the anus and perianal skin of landrace pigs, and the ultrasonic probe of the digital color ultrasonic diagnostic apparatus (G50, VINNO, China) was placed on the bare skin to observe the tissue morphology.

### 2.6. Histological Analysis

The tissues were fixed in the fixing solution (G1101, Servicebio, China) for more than 24 hours, and then the tissues were successively dehydrated and embedded. The wax block was placed in microtomes (RM2016, Leica, China) and cut into 4 *μ*m slices, which were then baked in a 60°C oven. The paraffin sections were subsequently dewaxed and hydrated, followed by staining with hematoxylin (G1004, Servicebio, China). After being subjected to differentiation and bluing, the sections were stained with eosin (G1001, Servicebio, China) and subjected to dehydration and transparency treatment. Thereafter, the sections were sealed with neutral balsam (10004160, Sinopharm Chemical Reagent Co., Ltd., China) and were observed under microscopy (Nikon Eclipse E100, Nikon, Japan).

### 2.7. ELISA Assay

The tissue was homogenized and centrifuged, and the supernatant was collected as an ELISA sample. The contents of EGF, platelet-derived growth factor (PDGF), vascular endothelial growth factor (VEGF), angiotensin- (Ang-) II, insulin-like growth factor- (IGF-) 1 receptor (R), transforming growth factor- (TGF-) *β*1, IL-6, IL-12, IL-1*β*, and TNF-*α* were measured with ELISA kits (Meimian, China). In brief, the sample was added to an ELISA plate which was coated with antibodies. After incubating for 30 minutes at 37°C, the sample was reacted with an enzyme-labeled antibody for 30 minutes at 37°C. Subsequently, the substrate chromogenic solution was added to the ELISA plate for chromogenic reaction, and finally, the reaction was stopped by stopping solution. The optical density (OD) value was measured with a microplate reader (CMaxPlus, MD, China) at a wavelength of 450 nm.

### 2.8. Terminal Deoxynucleotidyl Transferase-Mediated Deoxyuridine Triphosphate Nick End Labeling (TUNEL) Staining

TUNEL staining was carried out with the help of the TUNEL Kit (G1507, Servicebio, China). In brief, paraffin sections were dewaxed, hydrated, and repaired with proteinase K solution. Then, the slices were incubated with Triton X-100 (G1204 Servicebio, China), and then with 3% hydrogen peroxide solution (216763, Merck, Germany). After that, the slices were reacted with TUNEL reaction solution and Streptavidin-HRP solution in turn, and then undergo DAB color development (G1212, Servicebio, China) and hematoxylin staining. After dehydration, transparentization, and mounting, the sections were finally observed under a microscope.

### 2.9. Western Blot Assay

Protein extraction was performed by RIPA lysis buffer (R0010, Solarbio, China), after which the protein concentration was quantified using a BCA protein kit (BI-WB005, SBJBIO, China). Afterwards, equal contents of protein (30 *μ*g) were fractionated by SDS-PAGE and then transferred into PVDF membranes (Millipore, USA). After being blocked with 5% skim milk for 2 h, the membranes were incubated with primary antibodies at 4°C overnight. Then, the membranes were washed three times and incubated with secondary antibodies for 1 h at room temperature. The information on antibodies was shown in Table S1. Visualization of protein expression was conducted by ECL reagent (abs920, Absin, China) on eZwest Lite Auto Imaging System (Genscript, USA), and GAPDH or *β*-actin was selected as a loading control.

### 2.10. Immunohistochemistry and Immunofluorescence Staining

The paraffin sections were dewaxed, hydrated, and placed in citrate buffer (BL604A, Biosharp, China) and then heated by microwave for antigen repair. Thereafter, sections were soaked in a 3% hydrogen peroxide solution for immunohistochemistry. After being blocked by 3% BSA, the sections were incubated with primary antibodies including anti-EGF, anti-FSP1/A100A4, anti-CD34, anti-P-ERK, and antivimentin. The next day, the sections were incubated with secondary antibody or fluorescent secondary antibody at room temperature for 1 hour. For immunohistochemistry, the sections were developed by DAB and stained with hematoxylin, while for immunofluorescence, the sections were stained with DAPI (40728ES03, Yeasen, China). The sections were finally observed under a microscope or fluorescent microscope (NIKON ECLIPSE C1, Nikon, Japan).

### 2.11. Quantitative Reverse Transcription-Polymerase Chain Reaction (qRT-PCR)

Total RNA was extracted by TRIzol reagent (Sangon Biotech, China), subsequent to which reverse transcription was conducted using First Strand cDNA Synthesis Kit (abs601510, Absin, China) for cDNA generation. After that, cDNA amplified in a Real-Time PCR System (7500, ThermoFisher, USA) was traced by SYBR Premix Ex Taq^II^ Mix (Takara, Japan). The relative expressions were normalized to GAPDH using 2^−ΔΔCT^ approach [22]. The sequences of the primers are listed in Table S2.

### 2.12. 16S rRNA Sequencing

16S rRNA sequencing was conducted as previously described [22]. In brief, the DNA was separated from the sample by DNA kit (EE101-11, Transgen, China), then quantified by NanoDrop (ND-ONE-W, Thermo Fisher, USA). The target fragment was amplified and the product was purified by magnetic beads (N411-01, Vazyme, China) and then quantified by Quant-iT PicoGreen dsDNA Assay Kit (P7589, Thermo Fisher, USA). Next, sequencing libraries were prepared using TruSeq Nano DNA LT Library Prep Kit (20015964, Illumina, USA) and sequenced on the Illumina MiSeq/NovaSeq platform. Sequencing raw data were stored in FASTQ format and subsequently denoised or clustered by DADA2 [23] or VSEARCH [24]. QIIME 2[25] software was used for annotation, evaluation of alpha diversity of microorganisms, and principal coordinate analysis (PCoA). The amplicon sequence variant (ASV)/operational taxonomic unit (OTU) plots were produced using either the Venn Diagram package or the plotrix package to analyze species differences. Marker and intergroup differences were analyzed by linear discriminant analysis effect size (LEfSe), and metabolic pathways were analyzed by the KEGG and MetaCyc databases.

### 2.13. Cell Culture

Human skin fibroblasts (HSF) were purchased from Aybio (China) and cultivated in DMEM medium (SH30243.01, Hyclone, USA) with 10% fetal bovine serum (16140071, Gibco, USA) and 1% penicillin/streptomycin (15140148, Gibco, USA).

### 2.14. Transfection

HSF were seeded in 6-well plates (5 × 10^5^ cells/well) and cultured for 24 hours. ERK-specific small interfering RNA (siRNA) and its corresponding negative control (NC) were synthesized by Sangon (siRNA-ERK#1: TGGCTACGATGAGAACATGAACA; siRNA-ERK#2: GACCTTCAACCTCTATTACTATG; siRNA-ERK#3: CTCCTTTCTCCGGCAAAACGATG). The transfection was conducted with the help of a transfection reagent (L3000001; Thermo Fisher Scientific, USA). In brief, the reagent and siRNA were, respectively, diluted in the serum-free medium and then mixed, which was later added to cells for 24 hours of incubation. The interference efficiency was determined by Western blot.

### 2.15. Preparation of Human CGF

Venous blood was drawn from 3 healthy volunteers into anticoagulation tubes for the preparation of CGF as described previously [26]. All volunteers signed informed consent. In brief, the centrifugation procedures were as follows: 2700 rpm/min for 2 min, 2400 rpm/min for 4 min, 2700 rpm/min for 4 min, and 3000 rpm/min for 3 min. The middle layer of the centrifuged liquid was used for subsequent experiments.

### 2.16. Grouping

The cell experiments were conducted in three parts. First, HSF was incubated with different concentrations of human CGF (0, 5, 10, 15, 20, 25, and 30%) for 8 hours, and then the appropriate CGF concentration was screened by cell counting kit 8 (CCK8). Next, HSF was incubated with selected concentrations of CGF (0, 10, 15, and 20%) for 8 hours and the cells were tested for biological behavior. Third, cells transfected with siRNA-ERK or siRNA-NC were incubated with CGF (20%) for 8 hours and then evaluated for biological behavior.

### 2.17. CCK-8 Assay

HSFs were seeded in a 96-well plate (5 × 10^3^ cells/well) and cultured for 24 h. Next, cells were incubated with CCK-8 solution (HY-K0301, MCE, China) for 2 h, and a microplate reader was utilized to read the OD value at 450 nm.

### 2.18. Cell Proliferation Assay

Cell proliferation was determined by Yefluor 594 Edu Imaging Kits (40276ES60, Yeasen, China). Sterile coverslips were placed in 12-well plates and cells were subsequently inoculated in 12-well plates. EdU working solution was added to the cells and incubated for 4 h. The cells were fixed with 95% ethanol, washed with PBS, permeabilized with TritonX-100, and incubated with click reaction solution for 30 minutes. After that, the cells were stained with DAPI for 2 minutes and subsequently mounted. The image was photographed under a fluorescence microscope.

### 2.19. Scratch Assay

HSFs were seeded in a 6-well plate (5 × 10^5^ cells/well) and cultured for 24 hours. Linear wounds across the monolayer of cells were made by a pipette tip. After washing with phosphate-buffered saline (PBS, E607008, Sangon, China), the cells were cultured in the incubator for 24 hours. The scratch closure area was photographed at 0 and 24 hours using a microscope.

### 2.20. Statistical Analysis

The measurement data were presented as mean ± standard deviation. The measurement data conforming to normal distribution and homogeneity test of variance were analyzed using one-way ANOVA. LSD analysis was used for further pairwise comparison between groups. Dunnett's T3 test or independent sample *t*-test was used for heterogeneity of variance. Kruskal-Wallis *H* test was used for the measurement data does not conform to normal distribution. The statistical analysis was implemented with SPSS 16.0 software, with *P* < 0.05 considering statistical significance.

## 3. Results

### 3.1. CGF Treatment Promoted the Healing of Pig Fistula

**Figure 1:**
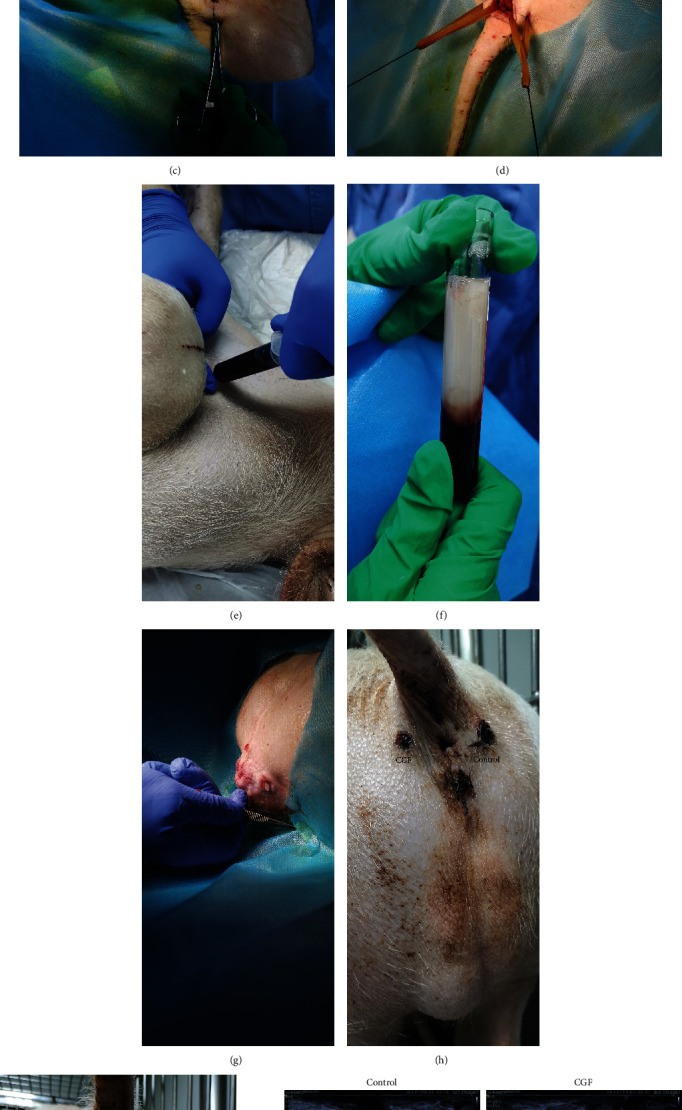
Porcine anal fistulae model creation process using rubber ligation surgery and treatment. (a) The marginal ear vessels injection with propofol at 2 mg/kg. (b) A 2 cm minimal incision was made in the anus at 3, 9, and 12 o'clock positions, and ligated rubber was used. (c) Directly cross through the anal rectal mucosa with the hemostatic clamp. (d) Rubber ligation was fixed to the fistula. (e) 10 ml blood sample was corrected from the pig. (f) Concentrated growth factor (CGF) was prepared from the pig itself. (g) CGF was used to treat the fistula at the 9 o'clock position. (h) CGF treatment for 2 weeks. (i) CGF treatment for 4 weeks. (j) B-mode ultrasound diagnosis imaging evaluation of created fistulas. (k) Posttreatment specimens of anal fistulae were stained with H&E and visualized at 20× and 40×.

Figures 1(a)–1(i) showed the process of anal fistula animal model construction and CGF treatment. Two and four weeks after CGF treatment, the pig fistula was examined by B-mode ultrasound. As shown in Figure 1(j), after two weeks of CGF treatment, a relatively broad lumen was visible in the fistula tissue of the control group, while the lumen in the fistula tissue of the CGF group was narrower than that of the control group and showed signs of healing. After four weeks of treatment with CGF, partial healing of the fistula was observed in the control group, but the hollow part was still clearly visible. The fistulas in the CGF group almost healed, and an elongated tube could be faintly seen.

In addition, we also evaluated the pathological changes of fistula tissue by H&E staining (Figure 1(k)). Four weeks after CGF treatment, in the control group, cicatrix repair was dominant in porcine fistula tissue wounds, and a small amount of granulation tissue was formed, accompanied by a large number of inflammatory cell infiltration, and no new capillary formation was observed. The wounds of porcine fistula tissue in the CGF group were repaired, and the inflammatory cell infiltration was reduced. The fibroblasts were mature and arranged neatly, and the distribution of new capillaries was visible.

### 3.2. CGF Increased Expressions of Angiogenesis-Related Factors and Inhibited Inflammatory and Apoptosis Levels in Granulation Tissue

**Figure 2:**
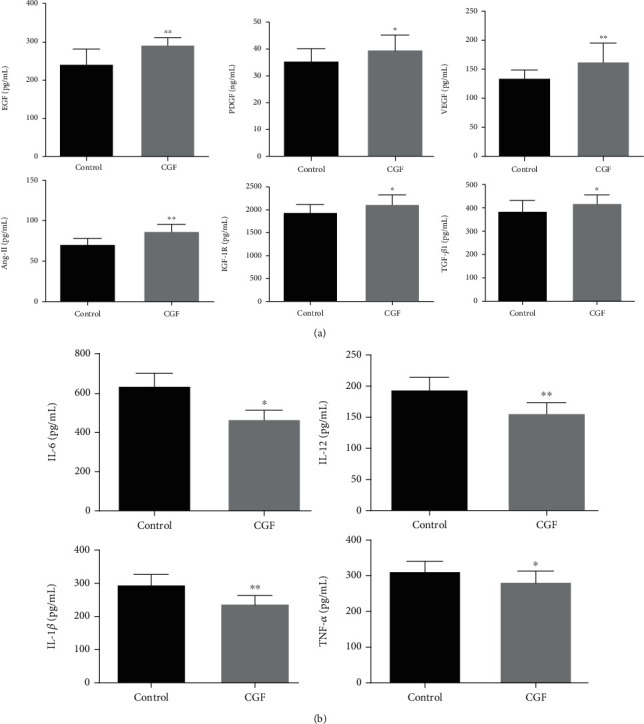
Effect of CGF on levels of vascular growth and inflammation-related factors in specimens of anal fistulae of pigs. (a) Levels of vascular growth-related factors EGF, PDGF, VEGF, Ang-II, IGF-1R, and TGF-*β*1 in specimens of anal fistulae were measured using ELISA assays. (b) Levels of inflammation-related factors IL-6, IL-12, IL-1*β*, and TNF-*α* were measured using ELISA assays. ^∗^*P* < 0.05, ^∗∗^*P* < 0.01 vs. control group.

**Figure 3:**
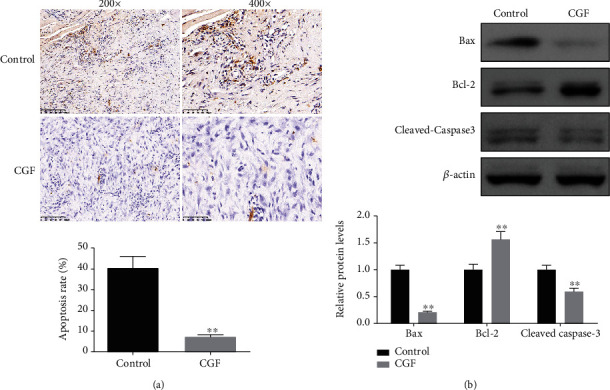
Effect of CGF on cell apoptosis of fistula tissues in pigs. (a) Cell apoptosis of fistula tissues was measured using TUNEL assay (scare bar = 50 *μ*m). (b) Protein expression levels of Bax, Bcl-2, and cleaved caspase-3 were detected by Western blot assay. ^∗^*P* < 0.05, ^∗∗^*P* < 0.01 vs. control group.

Wound blood supply and inflammatory response played an important role in wound healing, so we assessed the levels of angiogenesis-related factors and inflammatory factors in each group after 4 weeks of CGF treatment. We found that after treatment with CGF, the levels of angiogenesis-related factors (EGF, PDGF, VEGF, Ang-II, IGF-1R, and TGF-*β*1) in central granulation tissue of fistula wound were increased (Figure 2(a), *P* < 0.05), while the levels of inflammatory factors (IL-6, IL-12, IL-1*β*, and TNF-*α*) were decreased (Figure 2(b), *P* < 0.05). In addition, the TUNEL staining of central granulation tissue of fistula wound unveiled that CGF treatment reduced the apoptosis rate (Figure 3(a), *P* < 0.01). Besides, the results of Western blot showed that CGF suppressed the expression levels of Bax and cleaved caspase-3 and fostered the levels of Bcl-2 in central granulation tissue of fistula wound (Figure 3(b), *P* < 0.01).

### 3.3. CGF Promoted Fibroblast Activation and ECM Accumulation by Activating MEK/ERK Pathway

**Figure 4:**
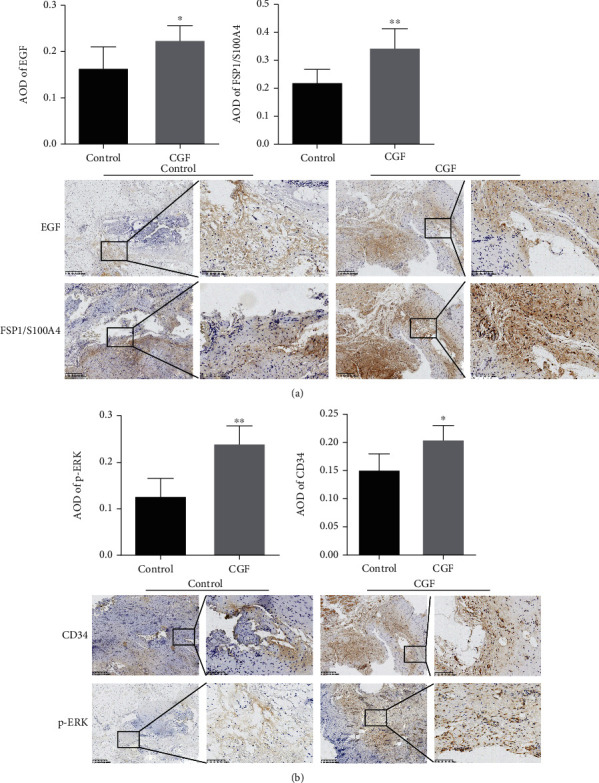
Effect of CGF on EGF, FSP1/S100A4, CD34, and p-ERK levels in fistula tissues of pigs. (a) EGF, FSP1/S100A4, (b) CD34, and p-ERK protein content in tissue samples were examined by IHC analysis. ^∗^*P* < 0.05, ^∗∗^*P* < 0.01 vs. control group.

**Figure 5:**
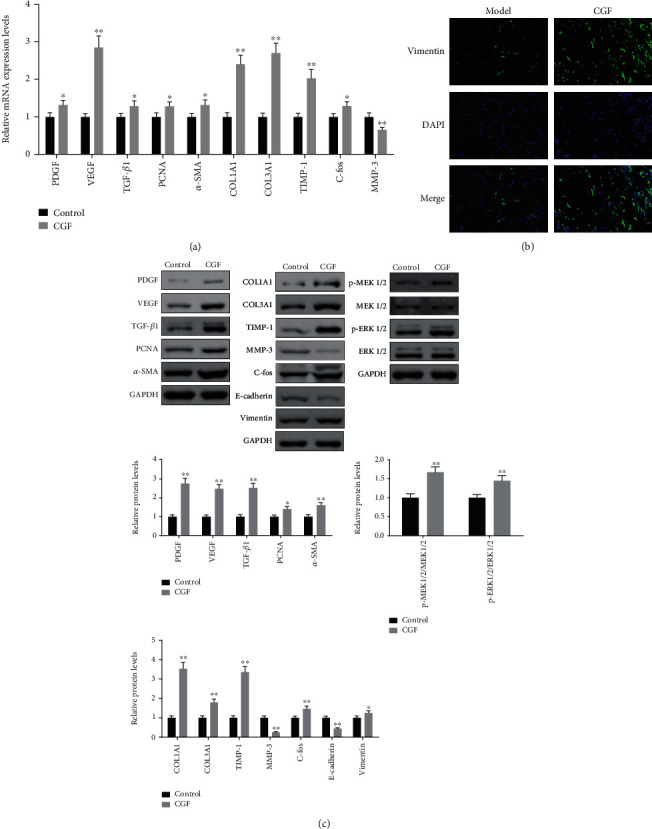
Effect of CGF on levels of healing-growth factors in fistula tissues of pigs. (a) The expression of PDGF, VEGF, TGF-*β*1, PCNA, *α*-SMA, Col1A1, Col3A1, TIMP-1, C-fos, and MMP-3 mRNA in tissue samples as examined by real-time PCR. (b) The protein levels of Vimentin were determined by immunofluorescence experiments. (c) The protein levels of PDGF, VEGF, TGF-*β*1, PCNA, *α*-SMA, Col1A1, Col3A1, TIMP-1, C-fos, E-cadherin, Vimentin, p-MEK1/2, MEK1/2, p-ERK1/2, and ERK1/2 were determined by immunoblotting. ^∗^*P* < 0.05, ^∗∗^*P* < 0.01 vs. control group.

We found that CGF treatment increased the expression of EGF, FSP1 (interstitial cell marker), CD34 (neovascular endothelial cell marker), and p-ERK in central granulation tissue of fistula wound (Figures 4(a) and 4(b)). Furthermore, qRT-CPR and Western blot experiments showed that factors related to the growth, proliferation, and differentiation of fibroblasts (PDGF, VEGF, TGF-*β*1, PNCA, and *α*-SMA) and genes related to the improvement of ECM function (COL1A1, COL3A1, TIMP-1, and C-fos) were upregulated by CGF, while the expression of MMP-3, a gene that inhibited ECM function, was downregulated by CGF (Figures 5(a)–5(c), *P* < 0.05). Our results also found that CGF also promoted vimentin but inhibited the expression of E-cadherin, indicating that the way of epithelial cells transform into fibroblasts for wound repair through EMT would be promoted by CGF (Figures 5(b)–5(c)). Meanwhile, CGF also promoted the expression of p-MEK1/2 and p-ERK1/2, which meant that CGF promoted the growth of fibroblasts by activating the MEK/ERK pathway (Figure 5(c)).

### 3.4. Analysis of 16S rRNA Sequencing

**Figure 6:**
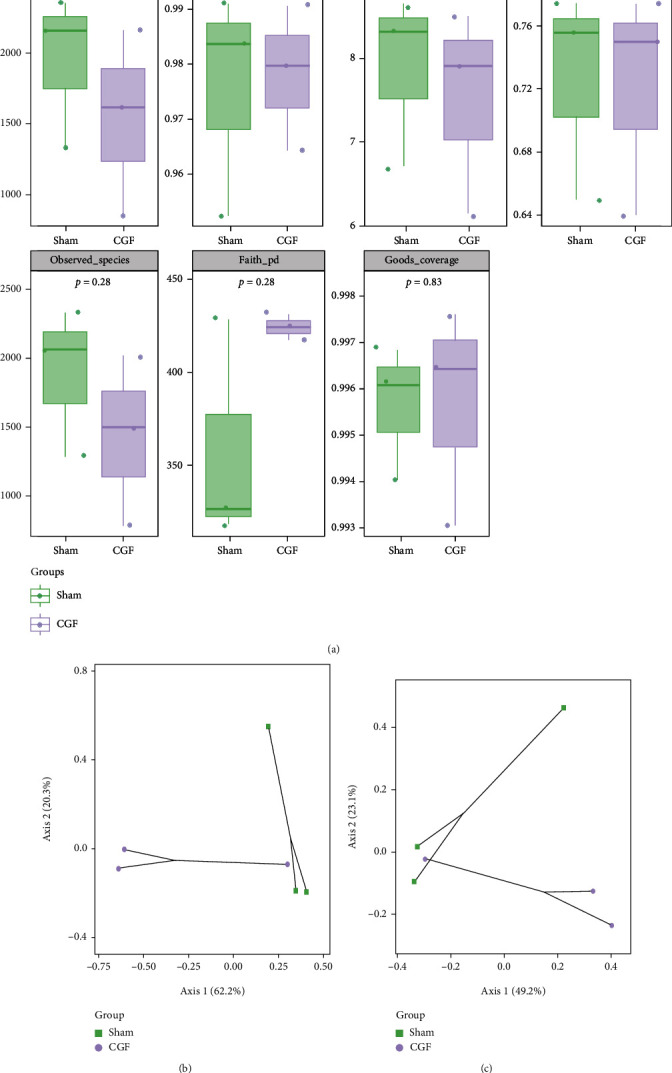
Gut microbial diversity in fistula tissues of pigs. (a) Alpha diversity was evaluated based on the Chao1, Simpson, Shannon, Pielou_e, Observed species, Faith_pd, and Goods_coverage indices of the OTU levels. Principal coordinates analysis of beta diversity was based on the weighted UniFrac (b) and Bray-Curtis (c) analyses of the OTU levels.

**Figure 7:**
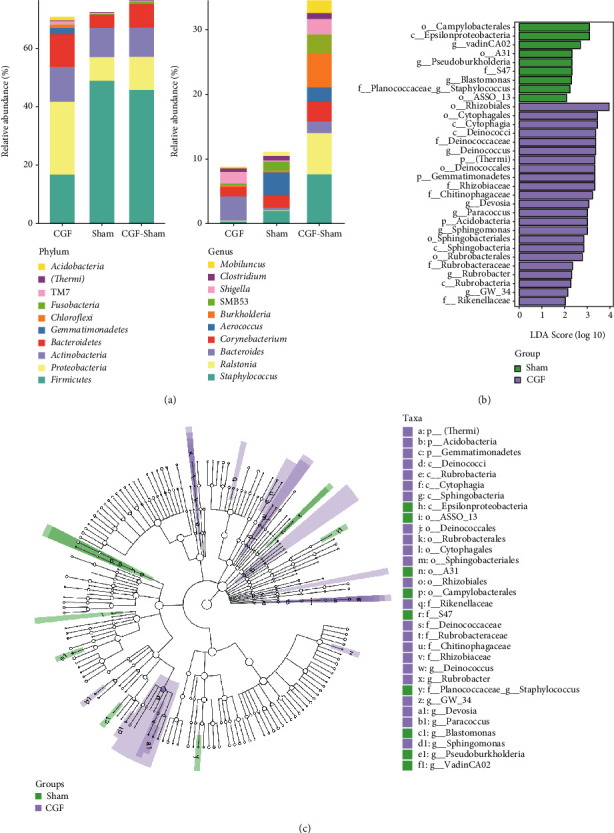
Composition of microbial communities from fistula tissues assessed by 16S rRNA gene sequencing. (a) Percent of community abundance on phylum level and genus level. *y*-axis represents relative abundance (%), *x*-axis represents each sample in the CGF group and sham group, and each taxonomic category is shown with a different color. CGF: CGF group, sham: control group. (b) Cladogram generated from the LEfSe analysis indicating the phylogenetic distribution from phylum to genus of the microbiota of CGF-treated pigs. (c) Histogram of LDA scores to identify differentially abundant bacterial genera between CGF-treated and control pigs.

**Figure 8:**
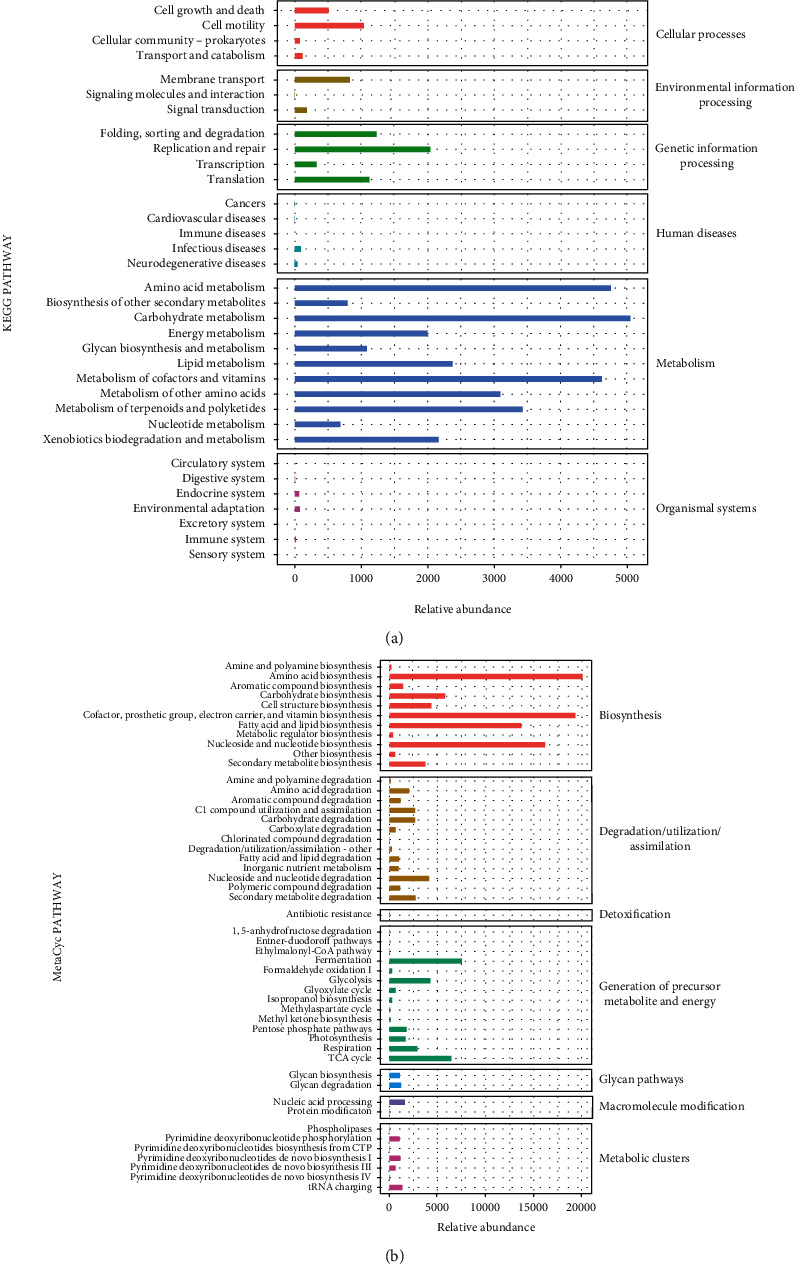
PICRUST2 analysis of the therapeutic role of CGF in fistula tissues of pigs. Phylogenetic Investigation of Communities by Reconstruction of Unobserved States (PICRUST2) based on 16S rRNA sequencing data was used to predict KEGG pathways (a) and metabolic pathway (b) of microbial communities.

To evaluate the effects of CGF on the porcine fistula microbiota, 16S rRNA sequencing was conducted on fistula samples. We assessed the alpha diversity between samples using 7 indices. Chao1 and Observed species characterized the richness of microorganisms. Simpson and Shannon indices indicated species diversity. Pielou_e indicated species evenness, Faith_PD indicated diversity based on evolution, and Goods_coverage indicated coverage. As shown in Figure 6(a), there are no significant differences in alpha diversity between the two groups (*P* > 0.05). Subsequently, PCoA was used to explore *β* diversity, and it can be seen that the two groups of samples were clearly distinguished (Figure 6(b) and 6(c)). To further analyze which species caused these differences, we counted the abundance of the corresponding ASV/OTU in each area at the level of phylum and genus and displayed it with a histogram (Figure 7(a)). The LEfSe analysis was then used to analyze significantly different species, with Figure 7(b) showing the predominant flora in the sham and CGF groups. According to the results of LEfSe, the microbial communities or species structures of different groups at different levels were shown by the cladogram. Seven different genera were identified in the fistula microbiota of the sham and CGF groups (LEfSe LDA > 2 and *P* < 0.05). *VadinCA02* and *Blastomonas* were increased in the sham group, whereas *Deinococcus*, *Devosia*, *Sphingomonas*, *Rubrobacteria*, and *GW_34* were increased in the CGF group (Figure 7(c)). The metabolic pathway analysis of the flora was carried out through the KEGG database and the MetaCyc databases. As illustrated in Figures 8(a) and 8(b), we found that the microbes were associated with amino acid and carbohydrate metabolism or biosynthesis, cofactor, prosthetic group, electron carrier, and vitamin biosynthesis, as well as nucleoside and nucleotide biosynthesis were more abundant in the CGF group. Furthermore, the CGF group had a higher abundance of microbes that participated in cell motility, translation, as well as replication, and repair.

### 3.5. CGF Promoted Cell Proliferation and Migration of HSF by Triggering the MEK/ERK Pathway

**Figure 9:**
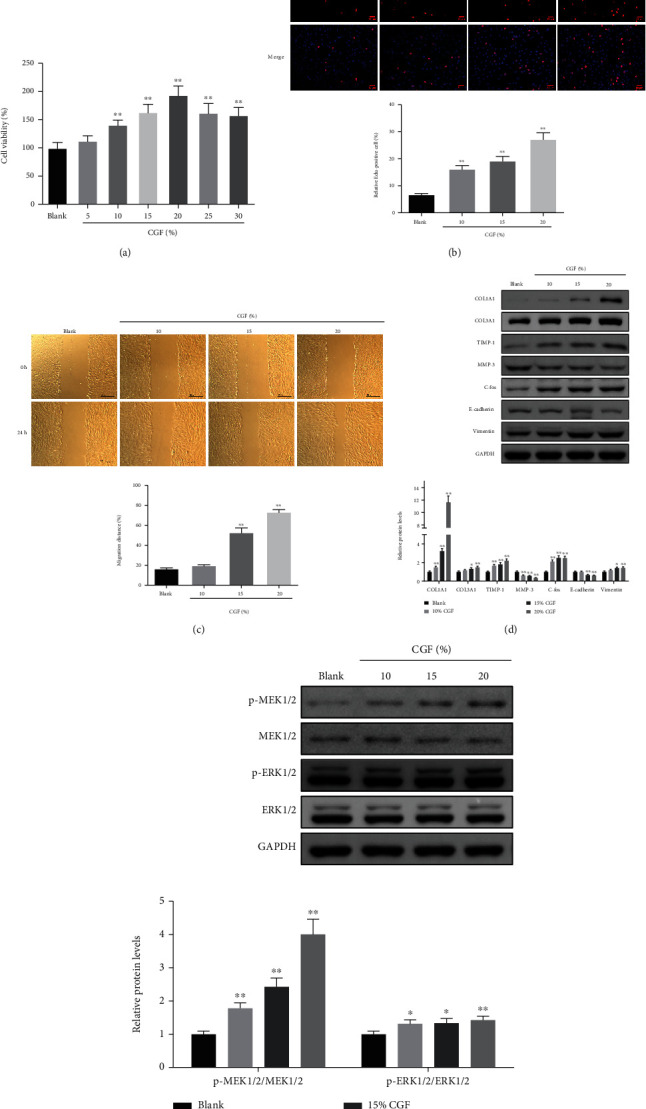
Effect of CGF on cell proliferation and migration ability by activating MEK/ERK signaling in HSF cells. (a) The effect of CGF on cell death in HSF cells at different dosages. (b) EdU assays of HSF cells treated with CGF were performed to evaluate cell proliferative ability. (c) Wound healing assays were performed in HSF cells treated with CGF at different dosages. (d, e) The protein levels of Col1A1, Col3A1, TIMP-1, MMP-3, C-fos, E-cadherin, vimentin, p-MEK1/2, MEK1/2, p-ERK1/2, and ERK1/2 to be measured after treatment of CGF in HSF cells by Western blot. ^∗^*P* < 0.05, ^∗∗^*P* < 0.01 vs. blank group.

To further validate the role of CGF in fistula wound healing, we conducted an in vitro experiment in which HSF was cultured with CGF extracted from the venous blood of healthy volunteers. CGF (5%, 10%, 15%, and 20%) promoted the viability of HSF, while CGF at concentrations above 20% (25% and 30%) was less effective at promoting cell viability than CGF at 20% (Figure 9(a), *P* < 0.01). Therefore, we selected 10%, 15%, and 20% CGF for follow-up experiments. Through phenotypic experiments, we determined that these three concentrations of CGF promoted the proliferation and migration of HSF (Figures 9(b) and 9(c), *P* < 0.01). Moreover, CGF can also regulate the expression of ECM function-related, EMT-related, and MEK/ERK pathway-related proteins in HSF, as shown in the fact that CGF resulted in the increase of COL1A1, COL3A1, TIMP-1, C-fos, vimentin, p-MEK1/2/MEK1/2, and p-ERK1/2/ERK1/2 as well as the decrease of MMP-3 and E-cadherin (Figures 9(d) and 9(e), *P* < 0.05).

### 3.6. Knockdown ERK Reversed the Effects of CGF on Cell Proliferation and Migration in HSF

**Figure 10:**
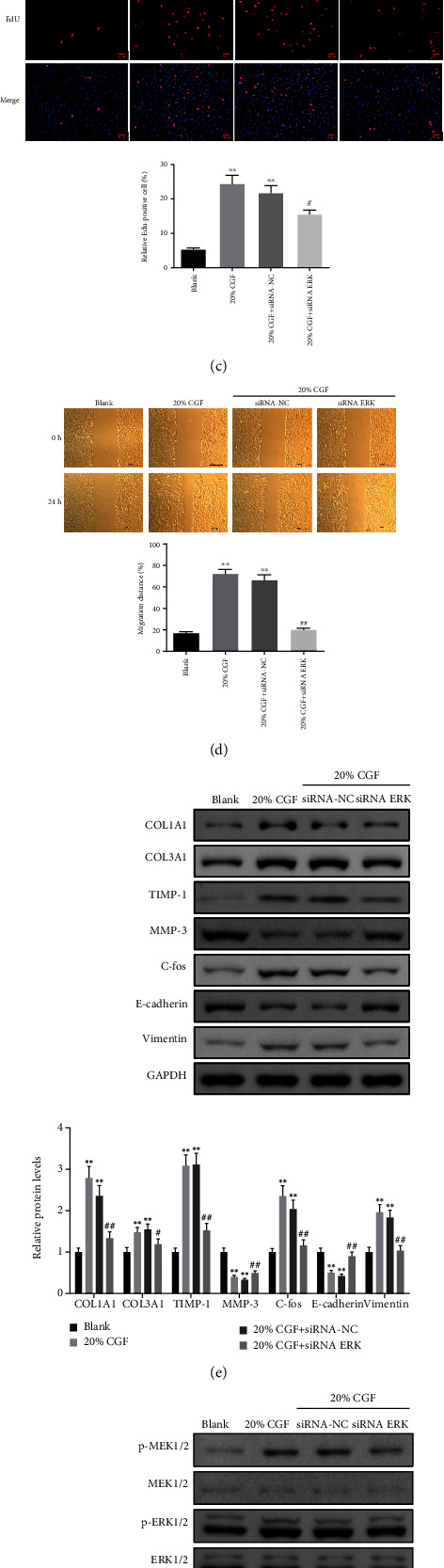
Knock-down of ERK effectively reverses CGF-induced activation of cell proliferation and migration ability in HSF cells. (a) Expression of ERK1/2 was confirmed by Western blot in HSF cells transfected with siRNA-NC or siRNA-ERK. (b, c) Cell proliferation was determined in 20% CGF-treated HSF cells following transfection with siRNA-ERK. (d) Cell migration assays were performed in 20% CGF-treated HSF cells following transfection with siRNA-ERK using wound healing assays. (e, f) The protein levels of Col1A1, Col3A1, TIMP-1, MMP-3, C-fos, E-cadherin, vimentin, p-MEK1/2, MEK1/2, p-ERK1/2, and ERK1/2 were detected in 20% CGF-treated HSF cells following transfection with siRNA-ERK by using Western blot assays. ^∗^*P* < 0.05, ^∗∗^*P* < 0.01 vs. blank group; ^#^*P* < 0.05, ^##^*P* < 0.01 vs. 20% CGF+siRNA-NC group.

Since the ERK pathway was closely related to wound healing [27], we used siRNA-ERK to assess whether the role of CGF in promoting wound healing was mediated by regulating the ERK pathway. We designed three siRNA-ERKs, among which the inhibition effect of siRNA-ERK#3 was the most significant (Figure 10(a), *P* < 0.05). Therefore, siRNA-ERK#3 was selected for subsequent experiments. Interestingly, we found that siRNA-ERK reversed the promotion effect of 20% CGF on the viability, proliferation, and migration of HSF (Figures 10(b)–10(d), *P* < 0.05). Furthermore, the regulation of 20% CGF on ECM function-related, EMT-related, and MEK/ERK pathway-related proteins were also reversed by siRNA ERK (Figures 10(e) and 10(f), *P* < 0.05).

## 4. Discussion

In this study, we reported the role of CGF in promoting wound healing in anal fistulas, consistent with previous reports that CGF promotes chronic wound healing [28]. As the anal fistula model established by small animals failed to explain the pathogenesis and related mechanisms of the anal fistula, we constructed the landrace pigs anal fistula model and then observed the effects of CGF treatment on the secretion of inflammatory factors, angiogenesis, fibroblast activation, ECM degradation, activation of the ERK pathway, and microbial population in pig fistula tissues to clarify the mechanism of CGF treatment for anal fistula healing.

Studies have reported that inflammatory factors and growth factors participated in wound healing. Specifically, overexpression of inflammatory factors, such as IL-6, IL-12, IL-1*β*, and TNF-*α*, causes slow wound healing by affecting fibroblast apoptosis and collagen expression. Growth factors, such as EGF, PDGF, VEGF, IGF-1R, TGF-*β*1, and Ang-II, promote the division and proliferation of fibroblasts and endothelial cells which migrate to the wound by inducing functional vascular growth, thereby improving the speed of wound healing [29]. Previous studies have proved that CGF contains high concentrations of growth factors and rich CD34 positive cells and can reduce the secretion of inflammatory factors, which were beneficial to wound healing [30, 31]. Consistently, our results found that CGF upregulated the expression of growth factors and downregulated the expression of inflammatory factors in porcine fistula, indicating that CGF can promote fistula healing by promoting angiogenesis and limiting the inflammatory response.

Fibroblasts, vascular endothelial cells, and epithelial cells are required to participate in the process of wound healing [32]. Epithelial cells differentiate into new fibroblasts through EMT, which express the mesenchymal marker FSP1 and can migrate to wounds to enhance ECM function by promoting collagen synthesis [33]. Fibroblasts then differentiate into myofibroblasts, which promote wound closure through *α*-SMA-mediated contraction [33^–^35]. Bonazza et al. reported that CGF can stimulate the growth and proliferation of fibroblasts and vascular endothelial cells [36]. Similarly, we found that CGF elevated the expressions of PCNA and C-fos associated with cell proliferation and inhibited the apoptosis-related protein in granulation tissue or HSF. In addition, Lei et al. pointed out that CGF could promote the migration of gingival mesenchymal stem cells and increase the expression of collagen, thus promoting gingival regeneration [37]. Shao et al. reported that a CGF-rich fibrin scaffold could promote collagen deposition and accelerate the healing of skin defects by promoting the secretion of type I and type III collagen, increasing the expression of TIMP-1 (MMP inhibitor) and inhibiting MMPs [38]. Consistent with the previous studies, our results have demonstrated that CGF promotes HSF migration, increases the protein levels of COL1A1, COL3A1, and TIMP-1 and inhibits the protein level of MMP-3. Although there is no direct evidence that CGF regulates cell EMT, platelet-derived biomaterials have been proved to improve bone injury by promoting EMT and enhancing the migratory ability of embryonic fibroblasts [39]. In this study, CGF, also a platelet-derived biomaterial, was able to promote vimentin expression and inhibit E-cadherin expression in granulation tissue and HSF, indicating that CGF accelerated wound healing by promoting the EMT process. Taken together, this evidence shows that CGF can enhance tissue repair by promoting fibroblast activation and inhibiting ECM degradation.

Growing evidence suggests that MEK/ERK pathway can induce cell proliferation and survival according to the various specific extracellular stimuli. CGF contains a large amount of EGF, and the binding of EGF to EGFR will lead to the activation of the downstream MEK/ERK pathway involved in the regulation of cell proliferation and metastasis [39, 40]. Furthermore, the pharmacological mechanism of some drugs for wound healing is associated with the activation of the MEK/ERK pathway [41, 42]. In particular, Yang et al. reported that plant extracts promoted wound healing of anal fistula by activating the MEK/ERK pathway [43]. Evidence has also shown that PRP can accelerate the repair of damaged musculoskeletal tissues by activating the ERK pathway [44]. In the present study, we found that p-MEK1/2 and p-ERK1/2 expressions were markedly upregulated in fistula tissues. Moreover, CGF treatment significantly promoted the proliferation and migration of HSF. Of note, ERK1 and ERK2, the bonafide substrates of MEK1/2, are supposed as the focal point of the MEK/ERK pathway to activate or inactivate a variety of proteins via phosphorylation in different subcellular compartments for inducing cell proliferation or cell cycle arrest [45]. Based on this, we knocked down the expression of ERK1/2 expression in CGF-treated HSF and assessed its effect on cell viability, proliferation, and migration. Our results suggest that the effect of CGF on proliferation and migration of HSF was reversed by ERK knockdown, which indicated that the effect of CGF on fistula healing was realized by activating the ERK pathway.

We analyzed the effect of CGF treatment on microbiota groups using 16S rRNA sequencing. After treatment with CGF, there was no significant change in species diversity, but microbial communities were significantly changed, as reflected in the increase of *Bacteroidetes* and *Bacteroides* and a decrease in *Firmicutes* and *Staphylococcus*. In particular, *Deinococcus*, *Devosia*, *Sphingomonas*, *Rubrobacteria*, and *GW_34* were increased in the CGF group. Previous studies have reported that the increase of *Bacteroidetes* and the decrease of *Firmicutes* are beneficial to intestinal health [46]. *Bacteroides* are generally beneficial in the intestine, whereas *Staphylococcus* promotes infection [47, 48]. Interestingly, Wang et al. found that *Bacteroides spp* that positively correlated with the progress of colitis was significantly increased in the gut, which decreased in the *Prunella vulgaris*- (PVH-) treated dextran sulfate sodium- (DSS-) induced acute colitis rats [49]. Altogether, our results show that the changes in the fistula microbiota may be related to the severity of fistula healing in anal fistula pigs. In addition, the KEGG and MetaCyc databases analyses revealed that these microbial communities may affect fistula healing through the metabolic and biosynthetic pathways of amino acid, carbohydrate, cofactors, and vitamins in this study. Therefore, the current results indicated that CGF treatment has created a healthy intestinal environment by changing the fistula microbial community. There are some limitations to the current study, including relatively small sample size and lacking clinical experiments. Also, the differential fistula microbes were obtained based on the 16S rRNA sequencing analysis. However, their role in fistula healing remains unclear. Therefore, CGF could be used for clinical therapy still needs a large number of clinical trials to be confirmed.

In conclusion, our research showed that CGF stimulated the MEK/ERK signaling pathway to suppress the inflammatory response, increase angiogenesis, fibroblast activation, and ECM function, as well as to control the microbiological environment of the fistula, so aiding in fistula repair. These results offer a theoretical foundation for the clinical use of CGF in minimally invasive anal fistula surgery.

## Data Availability

The datasets used and/or analyzed during the current study are available from the corresponding author on reasonable request.

## Supplementary Materials

Supplementary 1 Supplementary Table S1: Antibodies used in this study.

Supplementary 2 Supplementary Table S2: Primers used in this study.
