# Supplementary material for: Autologous Bioactive Compound Concentrated Growth Factor Ameliorates Fistula Healing of Anal Fistula in a Pig Model and Promotes Proliferation and Migration of Human Skin Fibroblasts via Regulating the MEK/ERK Pathway
Source: Oxid Med Cell Longev. 2022 Oct 14;2022:7660118. doi: 10.1155/2022/7660118 (PMC9587676; doi:10.1155/2022/7660118)
Supplement: Supplementary 1 — Supplementary Table S1: Antibodies used in this study. [file 7660118.f1.doc]

**Table S1 Antibodies used in this study**

| **Name** | **Catalog** | **Dilution** | **Applications** | **Manufacturer** |
| --- | --- | --- | --- | --- |
| Bax | AF0120 | 1/1000 | Western blot | Affinity, USA |
| Bcl-2 | AF4700 | 1/1000 | Western blot | Affinity, USA |
| Cleaved caspase-3 | AF7022 | 1/1000 | Western blot | Affinity, USA |
| PDGF | AF0240 | 1/1000 | Western blot | Affinity, USA |
| VEGF | AF5131 | 1/1000 | Western blot | Affinity, USA |
| TGF-β1 | AF1027 | 1/1000 | Western blot | Affinity, USA |
| PCNA | AF0239 | 1/1000 | Western blot | Affinity, USA |
| α-SMA | AF1032 | 1/1000 | Western blot | Affinity, USA |
| COL1A1 | AF7001 | 1/1000 | Western blot | Affinity, USA |
| COL3A1 | AF5457 | 1/1000 | Western blot | Affinity, USA |
| TIMP-1 | AF7007 | 1/1000 | Western blot | Affinity, USA |
| MMP-3 | AF0217 | 1/1000 | Western blot | Affinity, USA |
| C-fos | AF5354 | 1/1000 | Western blot | Affinity, USA |
| E-cadherin | AF0131 | 1/1000 | Western blot | Affinity, USA |
| Vimentin | AF7013 | 1/1000 | Western blot | Affinity, USA |
| P-MEK1/2 | AF8035 | 1/1000 | Western blot | Affinity, USA |
| MEK1/2 | AF6385 | 1/1000 | Western blot | Affinity, USA |
| P-ERK1/2 | AF1015 | 1/1000 | Western blot | Affinity, USA |
| ERK1/2 | AF0155 | 1/1000 | Western blot | Affinity, USA |
| β-actin | AF7018 | 1/5000 | Western blot | Affinity, USA |
| GAPDH | ab8245 | 1/10000 | Western blot | Abcam, UK |
| goat anti rabbit | ab205718 | 1/2000 | Western blot | Abcam, UK |
| goat anti mouse | ab205719 | 1/2000 | Western blot | Abcam, UK |
| EGF | ABIN6994767 | 1/50 | Immunohistochemistry | Antibodies, Germany |
| FSP1 | ABIN2789990 | 1/50 | Immunohistochemistry | Antibodies, Germany |
| CD34 | ab81289 | 1/2500 | Immunohistochemistry | Antibodies, Germany |
| p-ERK | ABIN744713 | 1/50 | Immunohistochemistry | Antibodies, Germany |
| goat anti rabbit | ab97051 | 1/2000 | Immunohistochemistry | Abcam, UK |
| Vimentin | ab92547 | 1/1000 | Immunofluorescence | Abcam, UK |
| Goat Anti-Rabbit IgG H&L (Alexa Fluor® 488) | ab150077 | 1/200 | Immunofluorescence | Abcam, UK |
