# Supplementary material for: Autologous Bioactive Compound Concentrated Growth Factor Ameliorates Fistula Healing of Anal Fistula in a Pig Model and Promotes Proliferation and Migration of Human Skin Fibroblasts via Regulating the MEK/ERK Pathway
Source: Oxid Med Cell Longev. 2022 Oct 14;2022:7660118. doi: 10.1155/2022/7660118 (PMC9587676; doi:10.1155/2022/7660118)
Supplement: Supplementary 2 — Supplementary Table S2: Primers used in this study. [file 7660118.f2.doc]

**Table S2 Primers used in this study**

| **Gene** | **Forward Primer (5′-3′)** | **Reverse Primer (5′-3′)** |
| --- | --- | --- |
| Pig PDGF | TTTGCCCTTTTTGGCGTAGC | CACCCAGACTGCTCACTGTT |
| Pig VEGF | GACCAGAAACCCCACGAAGT | TTAACTCAAGCTGCCTCGCC |
| Pig TGF-β1 | CTCAGACCTGCCTCAGCTTTC | CACTGAGGCGAAAACCCTCTAT |
| Pig PCNA | CTGGTCCAGGGCTCTATCCT | AGGCTAACAAGACCAGATTTGA |
| Pig α-SMA | GCCGGAGAGCAGCAGAAA | CCTTCCACAGGGCTTCGTTT |
| Pig COL1A1 | CTCTGATTGGCTGGGGGAC | CAGGGCGACCAGGCTTTC |
| Pig COL3A1 | CTGGTCCCCCTGGTGTATCT | AGAATCACCCTTGCCTCCTG |
| Pig TIMP-1 | TGGTCATCAGGGCCAAGTTT | GGTCTGTCCACAAGCAGTGA |
| Pig MMP-3 | GTGTGGCAGTTTGCTCAAGT | AAGGAAGAGGTGGCCAAAATGA |
| Pig C-fos | CTCTCCTACTACCACTCACCG | TCGGGGTAGGTAAAGACGAA |
| Pig GAPDH | TCGGAGTGAACGGATTTGGC | AGCTTGACGAAGTGGTCGTT |
